# Administrative and Medical Staff

**Published:** 1915-10-23

**Authors:** 


### THE TERRITORIAL HOSPITAL.

### I.—Administrative and Medical Staff.

The administrative side of the birth of the Territorial hospital, and the sound success which has attended its institution, are among the most interesting facts resulting from the great upheaval caused by the war. Territorial hospitals work under the War Office, and their administration and the executive details have emanated from that source. If we were to enter into full particulars of the system and our copy fell into the hands of the Censor's gentleman who is the hero of the mutilation of Rudyard Kipling's line "The captains and the kings depart," this page would probably consist mainly of the deepest black ink. In consideration for our readers and for our own time, which is valuable in these strenuous days, we will therefore refrain from any such attempt.

It will, however, interest many readers, and be quite beyond the power of the Censor's gentleman, to note that in the Territorial Service which has grown up since the war the Territorial hospitals are broadly divided into two types. First there are those Territorial hospitals which have taken over and are working in Poor-Law or other institutional buildings formerly devoted to the treatment of the sick, who were transferred elsewhere when the Territorials took possession. In this group, which we will refer to as Class A, the staff ordered to take charge of Territorial buildings of this type, when they enter them find each fully or largely equipped, or, in other words, ready-made for hospital purposes. The work which devolves upon the staff in such conditions, though arduous, is relatively light compared with the duties and strain which fall upon the heads of the staff, both medical and nursing, who are commissioned to take over a converted lunatic asylum, especially where, as in the Leicester case, the buildings have been unoccupied and unused for a number of years, and the same conditions, *cæteris paribus*, apply to an entirely new hospital building or one of temporary structures. In the latter case, which we will call Class B, everything has to be selected and supplied, for an entire and full equipment is necessary, and this has to be obtained, organised, distributed, and the building got into a state of perfect order for the reception of patients. No one who has not had to face the arduous labour and to give the devoted attention required by the chief officials and all their staff in the preparation, equipment, and opening of a new hospital can adequately appreciate or indeed have the smallest idea of the labour and strain involved. Hence it has not unnaturally come to pass that the staff, from the highest to the lowest, of a Territorial hospital belonging to Class B refer to their friends, the staff of Class A hospital, as having manifestly been called upon to display less knowledge and administrative gifts than themselves because they have not had to exhibit qualities the exercise of which by the staff of Class B proves their special efficiency in the making of the Territorial hospital to which they are attached. In a word, Class B have had to make their hospital from top to bottom, whilst Class A had theirs handed over to them mainly, if not entirely, ready-made. In other words, if the war were to continue indefinitely, which God forbid, and the demand for more and more hospital accommodation for the wounded were to continue, it must happen that all workers, and especially the higher officials in Class B, would come to occupy the relatively higher position which is now claimed unofficially in the humorous rather than the practical sense.

### The Medical Staff.

The medical staff of most Territorial hospitals has been drawn from the members of the medical profession resident in the district in which the Territorial hospital is placed. Some of them are members of the honorary medical staff of the voluntary hospitals in the district, some are in general practice; all of them are given commissions in the R.A.M.C., with relative rank according to the position which each may hold in the hospital to which he is attached. They have zealously taken up their duties, many of which must have been strange and difficult to numbers of these gentlemen, and they have and are fulfilling those duties with commendable efficiency. It may be well to say that the difficulties of some who have been called upon to take a high position in the administrative sense in a Territorial hospital, where they have had much to learn, have not been inconsiderable, and it is satisfactory to note, so far as our experience has gone, that these gentlemen have acquired or are rapidly acquiring much knowledge which renders the discharge of their duties, especially in the case of those men who have administrative gifts, both acceptable and adequate.

### The Orderlies and their Limitations.

One continuous difficulty which affects the medical staff of a Territorial hospital, especially if it is situated in the provinces or far away from the centre, is the management and training of orderlies. The orderly is usually recruited locally. He is drawn from sections which may have sent relatively few members to the fighting ranks; he is usually quite untrained for the office, and it devolves upon the medical staff to teach and train him as far as possible in his duties. This training often presents considerable difficulties, and it is discouraged and handicapped by the fact that directly a zealous medical officer has gathered around him and thoroughly trained a staff of orderlies they are liable to be ordered elsewhere. To this probability may be attributed most of the difficulty which is evidently experienced in maintaining smart and hygienic efficiency in the upkeep and condition of the sanitary adjuncts of the wards and generally. There is often far too much aroma and untidiness in this section of a Territorial hospital, which may ill compare with the same section of a well-administered voluntary hospital.

